# The Prospective Registration of Clinical Trial Protocols: When Is a Health-Related Intervention Study Not a ‘Clinical Trial’?

**DOI:** 10.3390/nursrep16010034

**Published:** 2026-01-21

**Authors:** Daniel Bressington, Wachira Suriyawong, Noppamas Pipatpiboon, Richard Gray

**Affiliations:** 1 Faculty of Nursing, Chiang Mai University, Chiang Mai 50200, Thailand; 2 School of Nursing, La Trobe University, Melbourne, VIC 3086, Australia; r.gray@latrobe.edu.au

According to the WHO, a clinical trial is defined as “any research study that prospectively assigns human participants or groups of humans to one or more health-related interventions to evaluate the effects on health outcomes” [1]. The WHO also states that these studies should have a trial protocol registered on a publicly accessible registry [2].

As journal editors and reviewers, we commonly encounter situations where the authors of a research article reporting a health-related intervention study have not provided details that a trial protocol was correctly (prospectively, before the first participant was recruited) registered (a requirement for *Nursing Reports* and most other reputable peer-reviewed scientific journals). When we request this information, we are often informed (disappointingly) that their ethics committee have not deemed the study to fall under clinical trial regulations, and therefore, trial registration was not required.

Excuses given by authors for not registering their trial are often somewhat opaque but tend to cluster around the following: “*it isn’t testing a device or medicine*”, “*it’s only a psychoeducational intervention*”, “*it’s a mixed methods study*”, “*it’s a pilot study*”, “*it was deemed low risk*”, “*only behavioural outcomes are measured*”, “*it’s a quasi-experimental study*”, “*it’s a health promotion intervention*”, “*it only has one arm*”, “*it has no control group*”, and “*it isn’t randomised*”.

These are not sound scientific reasons for not prospectively registering a trial. The point of registering a trial protocol is not only regulatory; it primarily serves to minimise the risk of the selective reporting of outcomes, reduce waste, and determine adherence to the study protocol. We have previously discussed the importance of prospectively registering trial protocols [3] and the concerning tendency among nurse researchers to resist what is generally regarded as standard practice in other clinical disciplines [4].

Although, as editors, we are clear about the requirements for pre-registration, it is not always so clear to authors. This may be due to the conflicting definitions of a “clinical trial” that are used in different countries, which may not be consistent with those applied by journal editors that tend to be guided by the International Committee of Medical Journal Editors (ICMJE) [5] and the Committee on Publication Ethics (COPE) [6]. In our experience, non-CTIMPS (clinical trials involving a medicinal product) of behavioural, psychological or educational interventions are often the source of most confusion.

To provide some clarification, we have summarised the definitions of a “clinical trial” applied by key international regulatory bodies, particularly in relation to non-pharmacological intervention studies. At *Nursing Reports*, we closely follow guidance from the ICMJE and the COPE. Most leading journals tend to apply the ICMJE definition of a clinical trial, which is consistent with the WHO definition [5]. The broad definition of a trial applied by the WHO is intended to promote prospective trial registration and improve the transparency of reporting. Similarly, the ICMJE [5] aims to reduce reporting bias and encourage the use of CONSORT/TiDier reporting guidelines for all health-related intervention studies conducted with humans. The Committee on Publication Ethics (COPE) endorses WHO/ICMJE standards for registration, transparency, and ethical publication.

However, many international regulators (for example, the Food and Drug Administration [FDA], European Medicines Agency [EMA], Medicine and Healthcare Product Regulatory Agency [MHRA], and Therapeutic Goods Administration [TGA]) apply a definition of a clinical trial restricted only to drugs or devices. Essentially, non-CTIMPs are not considered clinical trials unless a regulated product is involved. If a regulator determines that a study of a psychoeducational intervention is not a *clinical* trial, it is understandable that the need for a prospectively registered protocol may be overlooked. But this requirement will not be ignored by most journal editors and can make publishing the findings increasingly challenging for authors.

Table 1 contrasts the different definitions of a clinical trial across some of the different regulatory bodies and organisations. This list is not exhaustive, so please check your own national requirements carefully and enquire with your institution’s research office and/or ethics committee.

One can observe from the above table where confusion arises regarding the need for trial registration, particularly if a researcher is conducting a non-pharmacological intervention study whilst only following their national regulatory guidelines. In circumstances where researchers are told by ‘official’ sources that they do not need to register the trial protocol, it is imperative that the distinction between registering a trial protocol solely for regulatory purposes and registering to improve the reporting and conduct of trials is clear.

Researchers should be mindful that, for publication purposes and in the interest of transparency, they should assess their study in accordance with the WHO/ICMJE definitions of a trial. If their study meets the WHO/ICMJE criteria, a trial protocol must be registered, even if the local ethics committee do not require it. Several options for trial registration incur no cost, and most trial protocols can be easily copied from the original study protocol that was submitted for ethical approval and pasted into the registration website. Examples of appropriate (free) trial registry databases recognised by the WHO International Clinical Trials Registry Platform (ICTRP) [1] include ClinicalTrials.gov (USA) [15], the Australia and New Zealand Clinical Trial Registry (ANZCTR) [16] and the Chinese Clinical Trials Registry (ChiCTR) [17].

Figure 1 is a flowchart that can be used to determine if your study is deemed to be a clinical trial (for publication in *Nursing Reports* and all other journals following COPE/ICMJE guidelines).

Returning to our original question, “when is a health-related intervention study not a ‘clinical trial’?”, the answer is NEVER when following the COPE and ICJME requirements for journals. Before recruitment, register any study protocol that prospectively assigns human participants to one or more health-related interventions to evaluate health outcomes; otherwise, most journals (including *Nursing Reports*) will not consider it for publication.

## Figures and Tables

**Figure 1:**
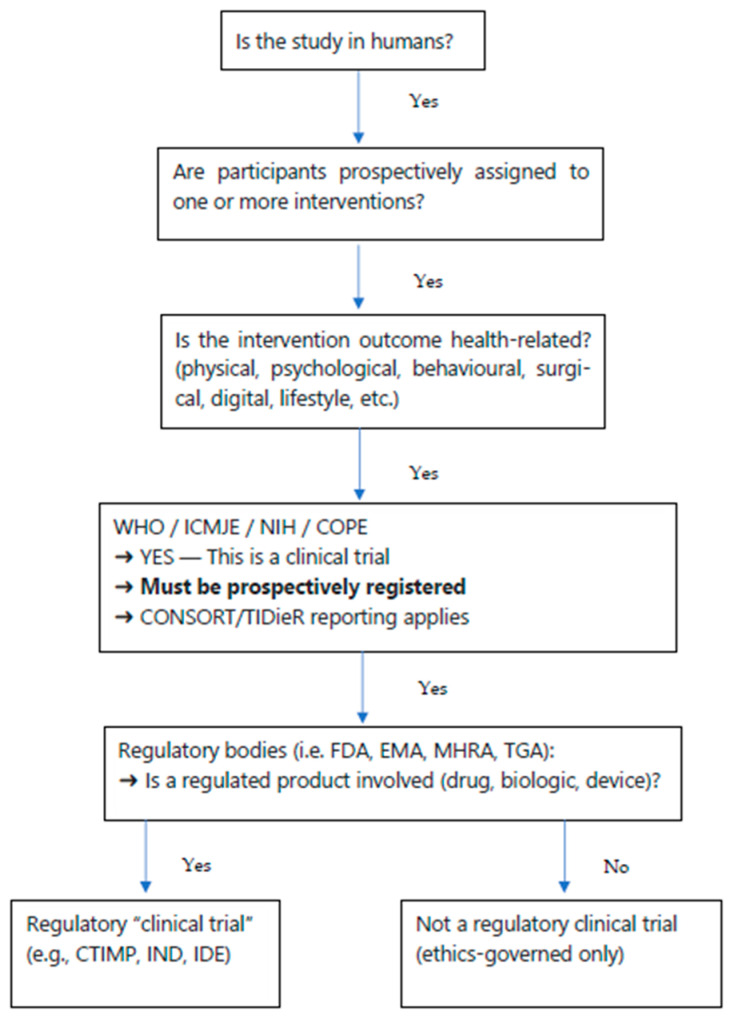
A flowchart for deciding if your study is deemed to be a clinical trial (for publication).

**Table 1:** Varying Definitions of Clinical Trials.

Organisation	Defines Clinical Trial?	Behavioural/Psychological Interventions Included?	Purpose	Notes
WHO (ICTRP) [6]	Yes	Yes	Global registration	Broad definition, all interventions included
ICMJE [3]	Yes	Yes	Publication standards	Requires prospective registration
COPE [4]	No	Yes (via WHO/ICMJE)	Publication ethics	No standalone definition
NIH (USA) [7]	Yes	Yes	Funding policy	Matches WHO closely
FDA (USA) [8]	Yes (narrow)	No	Product regulation	Only drugs/devices
EMA (EU) [9]	Yes (narrow)	No	Medicinal product regulation	
MHRA (UK) [10]	Yes (narrow)	No	Drug trials (CTIMPs)	
TGA (Australia) [11]	Yes (narrow)	No	Drugs/devices	Only drugs/devices
CIHR (Canada) [12]	Yes	Yes	Funding policy	Includes process-of-care changes, preventive care, and psychotherapies
TCRC (Thailand) [13]	No	Yes	Research transparency, redundancy, publication bias	Both clinical trials and observational studies
NMPA (China) [14]	Yes (narrow)	No	Drugs/devices	Only drugs/devices
